# Supplementary material for: The Mechanisms of BDNF Promoting the Proliferation of Porcine Follicular Granulosa Cells: Role of miR-127 and Involvement of the MAPK-ERK1/2 Pathway
Source: Animals (Basel). 2023 Mar 21;13(6):1115. doi: 10.3390/ani13061115 (PMC10044701; doi:10.3390/ani13061115)
Supplement: Supplementary file 1 [file animals-13-01115-s001.zip › Figure S1. Porcine follicular granulosa cells identification and the expression of BDNF and TrkB in GCs..pdf]

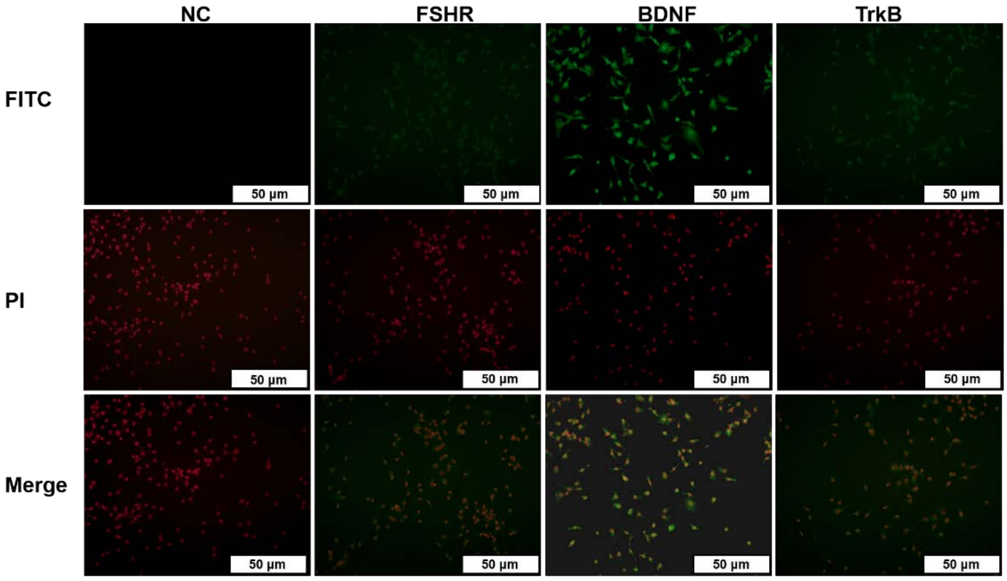

Figure S1. Porcine follicular granulosa cells identification and the expression of BDNF and TrkB in GCs. Top series, porcine GCs were incubated with primary antibody dilution buffer (NC), anti-FSHR, anti-TrkB or anti-BDNF, and then incubated with secondary antibody of FITC-conjugated (FITC, green). Middle series, the nuclei of GCs were located with PI (PI; red). Bottom series, the merged images of two fluorescences. The porcine GCs were stained and displayed two colures. The calculated mean percentage of positive cells with FSHR expression was 97.8±1.3% (data shown as mean ± SD, n > 200 cells per sample, N = 3) and according to the green fluorescence, BDNF and TrkB were abundantly expressed. Scale bar = 50 μm. NC, negative control; FSHR, follicle-stimulating hormone receptor; BDNF, brain-derived neurotrophic factor; TrkB, tyrosine kinase receptor B; FITC, fluorescein isothiocyanate; PI, propidium iodide.
