# Supplementary material for: The Mechanisms of BDNF Promoting the Proliferation of Porcine Follicular Granulosa Cells: Role of miR-127 and Involvement of the MAPK-ERK1/2 Pathway
Source: Animals (Basel). 2023 Mar 21;13(6):1115. doi: 10.3390/ani13061115 (PMC10044701; doi:10.3390/ani13061115)
Supplement: Supplementary file 1 [file animals-13-01115-s001.zip › Table S1. Primers used for qRT-PCR.pdf]

Table S1. Primers used for qRT-PCR

Primers	Sequence(5'-3')
BDNF-F	TTATTTCATACTTCGGTTGC
BDNF-R	CACTCGCTAATGCTGTCG
GAPDH-F	CCAGTATGATTCCACCCACG
GAPDH-R	ATTTGATGTTGGCGGGAT
Bcl-2-F	CTCAGCAACCCAGCAAACAC
Bcl-2-R	CTTGTTTTCCACTGCGGGTG
p21-F	CAGGACTGAGATGCACTGAT
p21-R	ACACGTTCCCAGGCGAAGT
CCND1-F	CACGACTTCATCGAGCACTT
CCND1-R	GTTTGCGGATGATCTGTTTG
Bax-F	TCTACCAAGAAGTTGAGCGAGTGT
Bax -R	CATCCTCTGCAGCTCCATGTTA
Dicer 1-F	CTAACGGCAGACTGGACG
Dicer 1-R	GAAGGGAGCAAGAGCAAT
ssc-miR-127-RT	GTCGTATCCAGTGCAGGGTCCGAGGTATTCGCACTGGATACGAC
ssc-miR-127-F	TCGGATCCGTCTGAGCTTGGCT
ssc-miR-185-RT	GTCGTATCCAGTGCAGGGTCCGAGGTATTCGCACTGGATACGAC
ssc-miR-185-F	GGAGAGGTTGCGAGTTCCTGAT
ssc-miR-1273-RT	GTCGTATCCAGTGCAGGGTCCGAGGTATTCGCACTGGATACGAC
ssc-miR-1273-F	AGACCACTGCAGCCTGGG
ssc-miR-7047-RT	GTCGTATCCAGTGCAGGGTCCGAGGTATTCGCACTGGATACGAC
ssc-miR-7047-F	GGAGGGCTGGGTCTGTGC
ssc-miR-532-RT	GTCGTATCCAGTGCAGGGTCCGAGGTATTCGCACTGGATACGAC
ssc-miR-532-F	ACACTCCCTCCCACACCCAAGG
ssc-miR-R	CAGTGCAGGGTCCGAGGTAT
U6 RT	AACGCTTCACGAATTTGCGT
U6 F	CTCGCTTCGGCAGCACA
U6 R	AACGCTTCACGAATTTGCGT
